# Novel simian foamy virus infections from multiple monkey species in women from the Democratic Republic of Congo

**DOI:** 10.1186/1742-4690-9-100

**Published:** 2012-12-05

**Authors:** William M Switzer, Shaohua Tang, Steve Ahuka-Mundeke, Anupama Shankar, Debra L Hanson, HaoQiang Zheng, Ahidjo Ayouba, Nathan D Wolfe, Matthew LeBreton, Cyrille F Djoko, Ubald Tamoufe, Amandine Esteban, Walid Heneine, Martine Peeters, Linda L Wright, Jean Jacques Muyembe-Tamfum, Emile Okitolonda Wemakoy, Prime Mulembakani, Nicole A Hoff, Anne W Rimoin

**Affiliations:** 1 Division of HIV/AIDS Prevention, National Center for HIV/AIDS, Viral Hepatitis, STD, and TB Prevention, Centers for Disease Control and Prevention, Atlanta, GA, 30333, USA; 2 Institut de Recherche Pour le Développement, Montpellier, France; 3 Institut National de Recherche Biomedicale, Kinshasa, Democratic Republic of Congo; 4 Global Viral Forecasting Initiative, San Francisco, CA, 94104, USA; 5 Eunice Kennedy Shriver National Institute of Child Health and Human Development, Bethesda, MD, 20892, USA; 6 Kinshasa School of Public Health, University of Kinshasa, Kinshasa, Democratic Republic of Congo; 7 Department of Epidemiology, UCLA School of Public Health, University of California at Los Angeles, Los Angeles, CA, 90095, USA

**Keywords:** Simian foamy virus, Retrovirus, Zoonosis, Africa, Women, Transmission, Public health, Emerging

## Abstract

**Background:**

Zoonotic transmission of simian retroviruses in Central Africa is ongoing and can result in pandemic human infection. While simian foamy virus (SFV) infection was reported in primate hunters in Cameroon and Gabon, little is known about the distribution of SFV in Africa and whether human-to-human transmission and disease occur. We screened 3,334 plasmas from persons living in rural villages in central Democratic Republic of Congo (DRC) using SFV-specific EIA and Western blot (WB) tests. PCR amplification of SFV polymerase sequences from DNA extracted from buffy coats was used to measure proviral loads. Phylogenetic analysis was used to define the NHP species origin of SFV. Participants completed questionnaires to capture NHP exposure information.

**Results:**

Sixteen (0.5%) samples were WB-positive; 12 of 16 were from women (75%, 95% confidence limits 47.6%, 92.7%). Sequence analysis detected SFV in three women originating from Angolan colobus or red-tailed monkeys; both monkeys are hunted frequently in DRC. NHP exposure varied and infected women lived in distant villages suggesting a wide and potentially diverse distribution of SFV infections across DRC. Plasmas from 22 contacts of 8 WB-positive participants were all WB negative suggesting no secondary viral transmission. Proviral loads in the three women ranged from 14 – 1,755 copies/10^5^ cells.

**Conclusions:**

Our study documents SFV infection in rural DRC for the first time and identifies infections with novel SFV variants from Colobus and red-tailed monkeys. Unlike previous studies, women were not at lower risk for SFV infection in our population, providing opportunities for spread of SFV both horizontally and vertically. However, limited testing of close contacts of WB-positive persons did not identify human-to-human transmission. Combined with the broad behavioral risk and distribution of NHPs across DRC, our results suggest that SFV infection may have a wider geographic distribution within DRC. These results also reinforce the potential for an increased SFV prevalence throughout the forested regions of Africa where humans and simians co-exist. Our finding of endemic foci of SFV infection in DRC will facilitate longitudinal studies to determine the potential for person-to-person transmissibility and pathogenicity of these zoonotic retroviral infections.

## Background

Human immunodeficiency viruses types 1 and 2 (HIV-1 and HIV-2, respectively) are recent examples of pandemic retroviruses which originated from cross-species infections from SIV-infected chimpanzees and gorillas, and sooty mangabeys, respectively [1]. Human T-lymphotropic viruses types 1, 2, and 3 (HTLV-1, -2, and −3) have an ancient origin from simian T-lymphotropic virus (STLV)-infected nonhuman primates (NHPs), but contemporaneous cross-species infections also occur [2]. The historical zoonotic origin and pandemic potential of simian retroviruses raises public health concerns associated with transmission of additional primate retroviruses to humans.

Simian foamy viruses (SFVs) are ancient retroviruses that have co-speciated with their NHP hosts for at least 30 million years [3]. Once acquired, SFV infections persist but do not apparently cause disease in NHPs, though systematic studies to evaluate disease associations are lacking [4,5]. Various studies report that humans exposed to NHPs show increased infection with simian retroviruses, including SFV [6-12]. Although the total number of human infections with SFV is not large, populations at increased risk include zoo workers and animal handlers in North America and Asia, and Africans who hunt and butcher NHPs for bushmeat [6,7,11-13]. However, little is known about the geographic distribution of SFV beyond these populations. In addition, information on the health consequences of SFV infection and its ability to spread from person to person is limited to data from cross-sectional studies and follow-up of small numbers of infected people for only short periods [6]. One U.S. study following seven persons for a median of 2 years following identification of SFV infection reported no evidence of secondary transmission or disease associations [14].

Hunting and butchering of wild animals provides nutrition and economic sustenance in many countries across Africa. In Central and West Africa alone, approximately 3–5 tons of bushmeat are killed every year, of which almost 15% comes from NHPs ( http://www.bushmeat.org). In addition to hunting and consumption by locals, there is also increasing international trade of bushmeat. These socioeconomic and behavioral factors result in frequent and possibly dangerous exposure to NHPs with concomitant increased risk of infection with viral agents, including simian retroviruses [15-19]. 75% of all emerging infectious diseases are estimated to have a zoonotic origin, the majority (37%) being comprised of RNA viruses [15,16]. The Democratic Republic of Congo (DRC) is an ideal setting for studies on the cross-species transmission and emergence of viruses considering the high levels of biodiversity and wild NHPs, and the widespread exposure of rural villagers to primates by hunting and butchering. For example, recent studies have documented the presence of novel SIVs and STLVs in bushmeat in DRC, and thus hunters may be at increased risk for infection with these retroviruses [17]. In addition, high rates of HIV-1 genetic variability have been reported in DRC, suggesting DRC as the epicenter of the global HIV-1/M pandemic, and supporting further the broad retroviral diversity in the region [20-22]. Given the high viral diversity in DRC and the emergence of other simian retroviruses in sub-Saharan Africa, we screened a large population of rural DRC inhabitants for SFV infection. Detailed animal exposure and demographic data were also collected to enable epidemiological assessments.

## Results

### Study population characteristics

**Figure 1:**
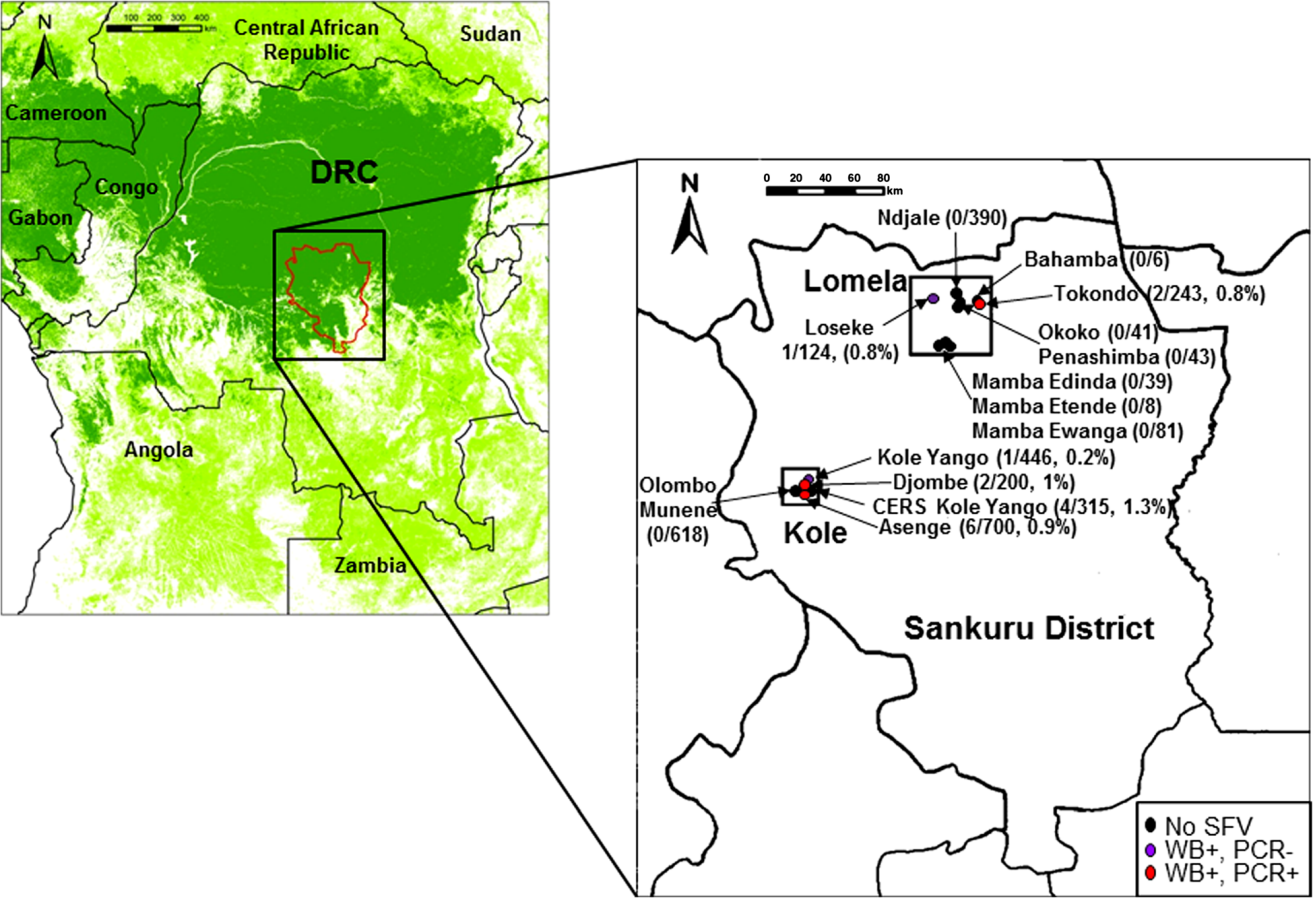
**Location of human infections with simian foamy virus (SFV) in the Democratic Republic of Congo (DRC).** Kole and Lomela health zones are shown in boxes with the 14 rural villages within the Sankuru district in the east central forested region of DRC. A key of the sites with different serologic and PCR evidence of SFV and those without evidence of SFV is provided. SFV prevalences are provided in parentheses after village names. Tree cover is indicated by light green, woodland; dark green, forest; white, savanna.

Of 3,846 consenting participants living in the Kole and Lomela health zones in 2007, demographic information on age, gender, and village was collected for nearly 97.5%. Villages in these two health districts included Asenge, Olombo Munene, Kole Yango, Ndjale, CERS Kole Yango, Tokondo, Djombe, Loseke, Mamba Ewanga, Mamba Edinda, Mamba Etende, Penashimba, Okoko, Penashimba, and Bahamba (Figure 1). Of these participants, approximately 58% were female, 84% were younger than 45 years of age, and 27% reported daily visits to the forest (Table 1). The average age was 24.2 yrs., with a median of 10 yrs. and range of 1 – 92 yrs. The age distribution was younger for males (median 15 yrs.) as compared to females (median 22 yrs.) (Wilcoxon rank sum test, p<0.05)

**Table 1:** Estimation of SFV prevalence by exposure to different simian species in DRC

					**Single variable models**		**Multivariable model**	
**Characteristic**	**# Exposed**	**Percent of population**	**# Infected**	**Prevalence (%)**	**Odds ratio**^**2 **^**(95% CL)**	**p-Value**	**Odds ratio**^**2 **^**(95% CL)**	**p-Value**
Female	2238	58.2	12	0.54	2.2 (0.7, 6.7)	0.18	1.8 (0.6, 5.7)	0.31
Male	1608	41.8	4	0.25	ref^3^		ref	
Age ≥ 45	635	16.5	5	0.79	2.3 (0.8, 6.7)	0.12	2.3 (0.8, 6.7)	0.13
Age < 45	3211	83.5	11	0.34	ref		ref	
Forest daily	1037	27.0	8	0.77	2.7 (1.0, 7.3)	<0.05	3.0 (1.0, 8.7)	<0.05
Forest <daily/never	2809	73.0	8	0.29	ref		ref	
Exposure^1^ to:								
Black mangabey	1781	46.3	12	0.67	3.5 (1.1, 10.9)	0.03	3.5 (0.8, 14.3)	0.09
No Black mangabey	2065	53.7	4	0.19	ref		ref	
Red-tailed guenon	1621	42.1	6	0.37	0.8 (0.3, 2.3)	0.71	0.6 (0.2, 2.2)	0.43
No red-tailed guenon	2225	57.9	10	0.45	ref		ref	
DeBrazza’s monkey	1273	33.1	4	0.31	0.7 (0.2, 2.1)	0.49	0.6 (0.2, 2.6)	0.50
No DeBrazza’s monkey	2573	66.9	12	0.47	ref		ref	
Agile mangabey	697	18.1	4	0.57	1.5 (0.5, 4.7)	0.48	1.2 (0.2, 5.9)	0.84
No agile mangabey	3149	81.9	12	0.38	ref		ref	
Wolf’s guenon	977	25.4	4	0.41	1.0 (0.3, 3.1)	0.97	1.2 (0.3, 5.1)	0.78
No Wolf’s guenon	2869	74.6	12	0.42	ref		ref	
Chimpanzee	498	13.0	2	0.40	1.0 (0.2, 4.2)	0.96	0.5 (0.1, 3.2)	0.46
No Chimpanzee	3348	87.0	14	0.42	ref		ref	
Angolan colobus	1265	32.9	10	0.79	3.4 (1.2, 9.4)	0.02	4.0 (1.1, 14.7)	0.04
No Angolan colobus	2581	67.1	6	0.23	ref		ref	
Gorilla	453	11.8	3	0.66	1.7 (0.5, 6.1)	0.39	2.6 (0.5, 13.9)	0.27
No Gorilla	3393	88.2	13	0.38	ref		ref	
Tshuapa’s red colobus	1390	36.1	5	0.36	0.8 (0.3, 2.3)	0.68	0.2 (0.1, 1.0)	>0.05
No red colobus	2456	63.9	11	0.45	ref		ref	
Unidentified NHP	2470	64.2	10	0.41	0.9 (0.3, 2.6)	0.89	0.7 (0.2, 2.2)	0.50
No Unidentified NHP	1376	35.8	6	0.44	ref		ref	

^1^Exposure behavior is discrete variable 0:6; primates with higher SFV prevalence (black mangabey, agile mangabey, Wolf’s guenon, Angolan colobus, gorilla) each represent 1 species-exposure, whereas the other five species groups represent 1-species-exposure collectively.

^2^The odds ratio is interpreted as 1 greater species-exposure. The descriptive percentage and estimated prevalence rates reflect any exposure to 1 or more species.

^3^ ref, reference group in regression models.

Individuals were asked about their frequency of entering forests, where animal diversity is greatest, to estimate possible animal contact. Self-reported encounters with NHP species included 1,781 (46%) persons who ever encountered black mangabey (*Lophocebus aterrimus*), 1,621 (42%) red-tailed guenon (*Cercopithecus ascanius*), 1,390 (36%) Tshuapa’s red colobus (*Piliocolobus tholloni*), 1,273 (33%) DeBrazza’s monkey (*Cerco*. *neglectus*), 1,265 (33%) Angolan colobus (*Colobus angolensis*), 977 (25%) Wolf’s guenon (*Cerco*. *wolfi*), 697 (18%), agile mangabey (*Cercocebus chrysogaster*), 498 (13%) chimpanzee (*Pan troglodytes*), and 453 (12%) gorilla (*G*. *gorilla*) (Table 1). 64.2% (2470/3846) of participants were not able to identify or remember one or more of the simian species they came in contact with. Exposure activities included 3,025 (79%) persons who ate 1 or more species of NHP, 2,195 (57%) cooked, 1,617 (42%) butchered or skinned, 247 (6%) hunted, 93 (2%) brought animals dead from the forest, 33 (1%) ate uncooked, 30 (1%) played with, and 8 (<1%) were scratched or bitten by NHP (Table 2).

**Table 2:** Estimation of SFV prevalence by type of activity with possible simian exposure in DRC

					**Single variable models**		**Multivariable model**	
**Characteristics**	**# Exposed**	**Percent of population**	**# Infected**	**Prevalence (%)**	**Odds ratio**^**2 **^**(95% CL)**	**p-Value**	**Odds ratio**^**2 **^**(95% CL)**	**p-Value**
Female	2238	58.2	12	0.54	2.2 (0.7, 6.7)	0.18	3.3 (0.8, 12.9)	0.09
Male	1608	41.8	4	0.25	ref^3^		ref	
Age ≥ 45	635	16.5	5	0.79	2.3 (0.8, 6.7)	0.12	1.9 (0.7, 5.7)	0.23
Age < 45	3211	83.5	11	0.34	ref		ref	
Forest daily	1037	27.0	8	0.77	2.7 (1.0, 7.3)	<0.05	2.4(0.9, 6.9)	0.10
Forest < daily /never	2809	73.0	8	0.29	ref		ref	
Exposure method^1^:								
Hunted	247	6.4	0	0	NA^4^		na	
Never	3599	93.6	16	0.45				
Brought animal dead from forest^5^	93	2.4	1	1.08	2.1 (1.3, 3.4)	0.003	2.2 (1.3, 3.9)	0.007
Never	3753	97.6	15	0.40	ref		ref	
Butchered, Skinned	1617	42.0	9	0.56	1.4 (1.1, 1.8)	0.02	1.4 (0.9, 2.1)	0.13
Never	2229	58.0	7	0.31	ref		ref	
Cooked	2195	57.1	10	0.46	1.2 (0.9, 1.6)	0.17	0.8 (0.5, 1.3)	0.36
Never	1651	42.9	6	0.36	ref		ref	
Ate	3025	78.6	12	0.40	1.2 (0.9, 1.5)	0.26	1.1 (0.7, 1.6)	0.82
Never	821	21.4	4	0.49	ref		ref	
Ate uncooked	33	0.9	0	0	NA		NA	
Never	3813	99.1	16	0.42				
Played with	30	0.8	0	0	NA		NA	
Never	3816	99.2	16	0.42				
Scratched or bitten by	8	0.2	1	12.50	2.8 (0.9, 9.0)	0.08	3.9 (1.2, 13.3)	0.03
Never	3838	99.8	15	0.39	ref		ref	

^1^Exposure behavior is discrete variable 0:6; primates with higher SFV prevalence (black mangabey, agile mangabey, Wolf’s guenon, Angolan colobus, gorilla) each represent 1 species-exposure, whereas the other five species groups represent 1-species-exposure collectively.

^2^The odds ratio is interpreted as 1 greater species-exposure. The descriptive percentage and estimated prevalence rates reflect any exposure to 1 or more species.

^3^ ref, reference group in regression models.

^4^ NA, not applicable. Exposure variables were not included in regression models if there were no cases among persons identified as having the exposure.

^5^Brought dead animal excludes hunting NHPs.

### SFV seroprevalence

**Figure 2:**
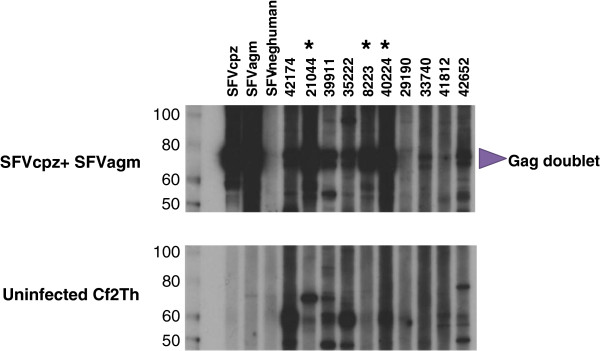
**Identification of human infection with simian foamy virus (SFV) in the Democratic Republic of Congo.** Detection of plasma antibodies to SFV from chimp (SFVcpz) and monkey (SFVagm) using a combined antigen Western blot assay. Upper panel shows reactivity to the combined antigen, lower panel shows reactivity to crude cell lysate antigens from uninfected canine thymocytes (Cf2Th). Seroreactivity was defined as those specimens with reactivity specific to the diagnostic Gag doublet proteins in the combined viral antigens. Lanes 1 and 2 show positive plasma controls from an SFV-infected chimpanzee and monkey, respectively. Lane 3 is a pedigreed negative human plasma control. Study participant plasma samples that were also PCR-positive are marked with an asterisk.

Plasma samples were available from 3,334 of the 3,846 participants (86.7%). 182/3,334 (5.4%) plasmas were reactive in the Luminex EIA, and 54/3,334 (1.6%) were also reactive in the standard EIA. 16/54 (29.6%) were WB seropositive, showing reactivity to the SFV Gag doublet proteins, giving an overall prevalence of 0.5% (16/3,334), 95% confidence limits (CL) (0.3 – 0.8). The WB assay has been previously shown to be 100% specific and sensitive for confirming infection with variant SFV [23]. Figure 2 is a representative of WB reactivity showing both seropositive and seronegative specimens.

### PCR and phylogenetic analysis of new SFV sequences

Buffy coats were available for PCR from 14/16 WB-positive persons. DNA integrity was confirmed in all 14 specimens by ß-actin PCR. DNA from only three persons (ID#s 8223, 21044, and 40244), all women, was positive for both SFV *pol* and LTR sequences (3/14, 21.4%). All three PCR-positive individuals showed strong WB positivity (Figure 2). DNA from the eleven other WB-positive persons was all negative for both LTR and *pol* sequences.

**Figure 3:**
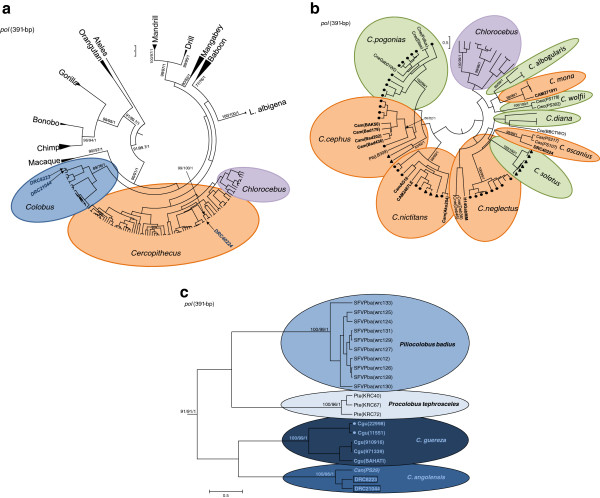
**Inference of the evolutionary history of human infections with simian foamy virus(SFV).a**. Circular maximum clade credibility (CMCC) tree of 173 SFV polymerase (*pol*) sequences generated by Bayesian analysis using the programs BEAST [44] and FigTree ( http://tree.bio.ed.ac.uk/software/figtree/) and a relaxed molecular clock. Trees were midpoint rooted. Final alignment length was 391-bp. Similar trees were inferred by maximum likelihood and neighbor-joining methods (data not shown). Inferred histories of the new human SFVs from DRC are shown with arrows. Major clades without human sequences from DRC are collapsed. **b**. Subtree of CMCC tree showing detailed phylogenetic relationships of human infections in the *Cercopithecus* and *Chlorocebus* clade. Dots and triangles indicate SFV sequences from nonhuman primates (NHPs) from Cameroon and Gabon, respectively. New SFV sequences from NHPs in DRC are in italics. SFV sequences identified in humans are in bold and country of origin is abbreviated (CAM, Cameroon; Gab, Gabon, DRC, Democratic Republic of Congo). Code for NHP species is first letter of genus followed by first two letters of species with animal name or code in parentheses, except for Ptt (*Pan troglodytes troglodytes*). **c**. Subtree of CMCC tree showing detailed phylogenetic relationships of human infection in the *Colobus* clade. New SFV sequence from *C*. *angolensis* from DRC is in italics. Dots indicate new SFV sequences from NHPs from Cameroon. SFV sequences identified in humans from DRC are in boxes. Code for NHP species is first letter of genus followed by first two letters of species with animal name or code in parentheses. Bootstrap support for NJ and ML analyses and posterior probabilities are given at selected nodes in that order. Reference sequences were obtained from GenBank.

To determine the primate origin of SFV infection in these three women, phylogenetic relationships were inferred by implementation of neighbor-joining, maximum-likelihood, and Bayesian methods using an alignment of *pol* sequences from 173 NHPs and humans. All three methods were highly congruent (data not shown). The majority of SFV sequences available from Africa originate from infected NHPs and humans residing in Cameroon; however, these sequences are limited to certain sampled species and do not include primates from DRC where our study population is located. Thus, to achieve the highest possible phylogenetic resolution we included in our analyses new SFV sequences from NHPs endemic to DRC (*Cercopithecus ascanius* (red-tailed guenon, n=2), *Ce*. *wolfii* (Wolf’s guenon, n=2), *Colobus angolensis* (Angolan colobus, n=1)), and new SFV sequences from NHPs hunted in Cameroon (*Ce*. *pogonias* (crested mona monkey, n=11), *Ce*. *cephus* (moustached guenon, n=5), *Ce*. *mona* (*Mona monkey*, *n*=*2*), *Ce*. *nictitans* (greater spot-nosed guenon, n=6), *Ce*. *diana* (Diana monkey, n=3), *Ce*. *neglectus* (DeBrazza monkey, n=8), *and Colobus guereza* (Eastern black and white colobus or mantled guereza, n= 4)). *Ce*. *neglectus* is present in both Cameroon and DRC. We also included recently reported SFVs from monkeys (*Ce*. *solatus* (sun-tailed guenon), *Ce*. *cephus*, *Ce*. *nictitans*) in Gabon, and humans infected with monkey SFVs from Gabon and Cameroon to increase phylogenetic resolution of the SFV evolutionary histories [11,12]. SFVs from different simian species clustered in statistically supported clades showing clear co-evolution of SFV with primate host (Figure 3a). Clades without DRC sequences were collapsed for clarity. Sequences from two women (8223 and 21044) clustered clearly with SFV from *Colobus* species with significant bootstrap and posterior probabilities (Figure 3a). The *pol* sequence from person 40224 clustered strongly within the *Cercopithecus*/*Chlorocebus* clade (Figure 3a).

When the subtrees containing the human DRC *pol* sequences are expanded visually to show each individual sequence, further resolution of the phylogenetic relationships and confirmation of co-evolution at the species level is revealed. Eleven distinct lineages within the *Cercopithecus*/*Chlorocebus* clade were inferred of which ten were *Cercopithecus* species-specific lineages, one contained the *Chlorocebus* SFV (Figure 3b). The *pol* sequence from person 40224 clustered strongly with *Ce*. *ascanius* (Figure 3b), while those from persons 8223 and 21044 clustered unambiguously with *Co*. *angolensis* with significant statistical support (Figure 3c). Both *Ce*. *ascanius* and *Co*. *angolensis* are endemic to DRC. The sequences from persons 8223 and 21044 shared 98.4% identity and were 96.7 – 97.4% identical to the *Co*. *angolensis* SFV *pol* sequence. The two *Ce*. *ascanius* sequences (PS217 and PS107) shared 99.3% identity, while the 40224 *pol* sequence shared about 95% nucleotide identity with the *Ce*. *ascanius* sequences.

Given the high levels of phylogenetic resolution at the species level in the *Cercopithecus* clade, we also included in our analyses *pol* sequences from infected humans in Cameroon and Gabon to explore further the species origin of these SFVs that were previously inferred to originate from cross-species infection from *Cercopithecus* species [11,12,24,25]. Seven sequences from infected Cameroonian hunters (AG16, Ako254, Bad50, Bad179, Bad202, Bad436, Cam2467LE) were previously thought to originate from *Ce*. *neglectus* by clustering with SFV from captive animals (Cne(Pollux) and Cne(Elise)). However, we show that SFV in Cameroonians AG16, Ako254, Cam2467LE are within the diversity of SFV from *Ce*. *nictitans* from Cameroon with strong statistical support (Figure 3b). These results are in agreement with recent reports that investigated the origin of SFV infection in a hunter from Gabon and Cameroon [11,12]. Four other hunter SFVs (Bad50, Bad179, Bad202, and Bad436) clustered strongly within the Cameroonian *Ce*. *cephus* clade (Figure 3b) and not *Ce*. *neglectus* as described previously [11]. Only SFV from an infected hunter in Gabon (H14GabMM) showed phylogenetic relatedness to SFV from *Ce*. *neglectus* from Kenya (Deb36 and Deb46), but with weak support (Figure 3b), and which contrasts with the finding from another study that showed it clustered with *C*. *nictitans* from Gabon. SFVs from the two captive (Pollux and Elise) and one wild *Ce*. *neglectus* from Gabon (CneGab01WD) all fell within the diversity of the *Ce*. *pogonias* SFV (Figure 3b), and not *Ce*. *neglectus* as originally inferred, indicating probable cross-species transmission with this virus while in captivity and in the wild. The two captive *Ce*. *neglectus* were in a mixed monkey coral, including *Ce*. *pogonias*, supporting this possibility, while the provenance of the wild DeBrazza was not reported [12].

Phylogenetic analysis of the LTR sequences was not performed since only a small number of SFV sequences from a limited number of simian species are available at GenBank. In addition, the LTR xregion is highly divergent, and thus alignments with all available SFVs do not contain enough phylogenetic signal to accurately resolve the genetic relationships. LTR sequences from persons 8223 and 21044 were 96.6% identical and shared 70.8 – 73.4% nucleotide identity with LTR sequences from *Co*. *guereza* (Cgu910916) and *Procolobus r*. *tephrosceles* (KRC6, KRC67, KRC70, and KRC72) [26]. The 40224 LTR sequence was nearly equidistant to those from various *Cercopithecus* SFVs, including *Ce*. *albogularis* (Cal(Syk119), *Ce*. *neglectus* (CNE01), *Ce*. *mona* (Cmo104), and the Cameroonian hunter infected with a *Ce*. *nictitans* SFV (Cam2467LE) [25], sharing between 80.1 – 89.1% nucleotide identity.

All new *pol* and LTR SFV sequences generated in the current study have been deposited in GenBank with the following accession numbers JX157538 – JX157580.

### Proviral loads in SFV-infected women

The new qPCR assay was found to be 100% specific by not detecting SFV in 76 blood donor and 10 commercially available human genomic DNAs all previously shown to be negative using nested generic *pol* PCR [23]. We also observed excellent sensitivity and broad detection of SFV variants from monkeys and apes. The assay detected 1–5 copies of SFVcpz(strains 199917 and X174), SFV1(mac), SFV3(agm), SFV10(bab), SFV11(ora), SFVmnd(1465MV), SFVcmo(2719YI), SFVdeb(2467LE), and SFVgor(1083MO). One woman (DRC21044) infected with Angolan colobus SFV had 1,755 copies/10^5^ cells, while proviral loads in the other two women (DRC8223 and DRC40224) were much lower (319 and 14 copies/10^5^ cells, respectively). DNA from the 11 other WB-positive persons tested below the limit of detection in the qPCR assay.

### Epidemiology

Demographic, exposure, and infection data of the SFV seropositive persons are summarized in Table 3. The ages of the 16 WB-positive persons ranged from 11 to 62 yrs. old (mean 32.7 yrs., median 27 yrs.); 12/16 (75%) were female. SFV infection was observed in six of the 14 villages in the Kole and Lomela health zones with prevalences ranging from 0.2 – 1.3% (Figure 1). Two WB-positive individuals did not report contact with NHPs though they reported entering the forests at least four times per month. Thirteen persons reported NHP exposure, including picking up carcasses for consumption, butchering and preparing for consumption, and eating. One person (23542) did not complete the questionnaire to provide primate exposure.

**Table 3:** Identification of frequent simian foamy virus (SFV) infection in rural DRC

**ID**	**Sex, Age**	**Village**	**Forest frequency**	**NHP exposure**^**1**^	**SFV WB**	**SFV *pol *PCR**^**2 **^**sequence**
6753	F,12	Tokondo	Never	B, PE, E	Positive	Negative
8223	F, 23	Tokondo	Everyday	B, PE, E	Positive	***Co. angolensis***
14092	M, 13	Loseke	2-4 times/month	PDE, B	Positive	Negative
21044	F, 57	Asenge	Everyday	PE, E	Positive	***Co. angolensis***
22492	F, 62	Olombo Munene	>4 times/month	B, PE, E, S, BN	Positive	NA
23542	M, 32	Asenge	2-4 times/month	NA	Positive	Negative
30015	F, 62	Asenge	Everyday	B, PE, E	Positive	Negative
32863	F, 36	Asenge	Everyday	B, PE, E	Positive	NA
33740	F, 13	Asenge	>4 times/month	B, PE, E	Positive	Negative
35222	F, 18	CERS Kole Yango	Everyday	None reported	Positive	Negative
38662	F, 21	CERS Kole Yango	>4 times/month	B, PE, E	Positive	Negative
39443	F, 11	CERS Kole Yango	Never	E	Positive	Negative
39911	M, 59	Djombe	Everyday	B, PE, E	Positive	Negative
40224	F, 50	Djombe	>4 times/month	None reported	Positive	***Ce.ascanius***
41274	F, 25	CERS Kole Yango	Everyday	PE, E	Positive	Negative
42652	M, 29	Kole Yango	>4 times/month	E	Positive	Negative

1.NHP, nonhuman primate; PDE, pick up dead monkey to eat; B, butcher monkey; PE, prepare monkey to eat; E, eat monkey; S, scratched by monkey; BN, bitten by monkey.

2.*pol*, polymerase; when PCR is positive monkey origin of infection is provided.

All three PCR-positive persons were women 23, 57, and 50 years of age from Tokondo, Asenge, and Djombe, respectively. Asenge and Tokondo are about 200 km apart and represent the greatest distance between villages in the study (Figure 1). Person 8223 reported exposure to Angolan colobus and black mangabey and is infected with Angolan colobus SFV (Table 3). Person 21044 reported exposure to red-tailed guenon but is infected with Angolan colobus SFV. Person 40224 reported no primate exposure but is infected with red-tailed guenon SFV and reported entering the forest at least four times per month (Table 3). Neither PCR positive woman who reported monkey exposure reported being bitten by monkeys, which contrasts with the increased level of bite wounds associated with SFV infection reported in other studies in Africa [11,24]. Buffy coats were not available from a WB-positive 62 year old woman (22492) and a 36 year old female (32863) for PCR testing.

Plasma specimens were available for WB testing from close contacts, defined as living in the same household, of person 21044 and from those of seven other WB-positive participants to evaluate person-to-person transmission of SFV (Table 4). The teenage daughter and son of person 21044 were both negative for antibodies to SFV. All 20 contacts of the seven other WB-positive participants were also WB negative, including four spouses, two parents, 5 offspring, and 6 siblings (Table 4).

**Table 4:** No evidence of transmission of simian foamy virus (SFV) to close contacts of Western blot-positive persons from the Democratic Republic of Congo

**ID of seroreactive person**	**Role in family**	**Sex, Age**	**ID of Relative**	**Relationship to seroreactive person**	**Sex, Age**^**1**^	**SFV EIA Luminex**
21044^2^	Head^3^	F, 57	29573	Daughter	F, 15	Negative
			29584	Son	M, 12	Negative
23542	Head	M, 32	23273	Wife	F, 41	Negative
			27075	Daughter	F, 5	Negative
30015	Wife of head (not primary)	F, 62	20274	Daughter	F, 10	Negative
			18233	Son	M, 8	Negative
			20335	2^nd^ wife of head (not primary)	F, 40	Negative
			30004	Grandson of head	M, 4	Negative
32863	Wife of head (primary)	F, 36	32826	Son	M, 8	Negative
			33574	Daughter	F, 10	Negative
			34090	Daughter	F, 6	Negative
33740	Daughter of head	F, 13	32992	Wife of head	F, 21	Negative
			33762	Maternal aunt	F, 23	Negative
			33773	Father (head)	M, 29	Negative
			33784	Brother	M, 7	Negative
			33751	Uncle	M, 21	Negative
35222	Daughter of head	F, 18	35200	Father (head)	M, 79	Negative
			35211	Brother	M, 6	Negative
			35233	Brother	M, 4.9	Negative
39443	Daughter of head	F, 11	39421	Wife (not primary)	F, 28	Negative
			39432	Sister	F, 16	Negative
			39454	Sister	F, 9	Negative
41274	Daughter of head	F, 25	41285	Brother	M, 24	Negative

1.Age in years.

2.Infected with SFV from *Ce*. *ascanius* (red-tailed guenon).

3.Head of household.

We analyzed the questionnaire data to determine if exposure to specific primates or activities increased the risk of SFV WB positivity. Using the multiply imputed complete datasets the estimated SFV prevalence was 0.42% (95% CL 0.24 - 0.67), which is slightly lower than the actual prevalence of 0.5% in the 3,334 participants for whom plasma was available for testing. Estimated SFV prevalence was significantly elevated for persons who frequented the forest daily (0.77%, 95% CL 0.33 - 1.51), who were exposed to black mangabey (0.67%, 95% CL 0.35 - 1.17) or Angolan colobus (0.79%, 95% CL 0.38 - 1.45), who brought dead NHPs home from the forest (1.08%, 95% CL 0.03 - 5.85), who butchered or skinned NHPs (0.56%, 95% CL 0.25 - 1.05), or who were scratched or bitten by NHPs (12.50%, 95% CL 0.32 - 52.65) (Table 1). These same exposures were significant (p<0.05) in the single-variable logistic regression models, with odds ratios (OR) of 1.4 and 2.1 (95% CL 1.1, 1.8 and 1.3, 3.4, respectively) for each additional species-exposure due to butchering and skinning NHP or bringing dead animals from the forest, respectively, and odds ratios of 3.4 and 3.5 (95% CL 1.2, 9.4 and 1.1, 10.9, respectively) for any type of exposure to Angolan colobus and black mangabey, respectively (Table 1). Because few persons were bitten or scratched by NHP, the association of this exposure with SFV prevalence was not statistically significant in the single-variable model, but this activity was independently associated with higher SFV prevalence in the multivariable model (OR 3.9, 95% CL 1.2 - 13.3). Other independent risk factors for SFV infection that were significant (<0.05) in the multivariable model include exposure to Angolan colobus (OR 4.0, 95% CL 1.1 - 14.7) and bringing dead NHPs from the forest (OR 2.2, 95% CL 1.3 - 3.9). Interestingly, making daily trips into the forest was significantly associated with risk for SFV infection as evidenced by the model that controlled for NHP species (OR 3.0, 95% CL 1.0 - 8.7) (Table 1), but was no longer significant and had reduced strength of association to SFV infection based upon results from the model that included explanatory variables for activities while in the forest (Table 2).

## Discussion

Human infection with SFV was first identified in 1971 in a Kenyan with nasopharyngeal carcinoma and since then has been found in other populations around the world who are exposed to NHPs, but without overt evidence of disease [6]. To date about 136 persons have been identified with SFV infection in 11 different countries on four continents [6,11,12]. However, given the high prevalence of SFV in NHPs, the frequent exposure to NHPs by persons who handle and consume NHPs, and the increasing demand for bushmeat in Africa and globally, models have predicted an even higher potential prevalence of SFV among humans [27]. Thus, to better assess the public health implications of this novel human infection requires a more comprehensive understanding of the epidemiology of SFV, including its geographical distribution, the evolutionary history of SFV in humans and NHPs, determination of exposure risks, and a thorough evaluation of secondary transmissibility and pathogenicity of SFV in humans and NHPs.

DRC is an ideal location for increasing our knowledge about emerging zoonotic infections since the majority of the population lives in rural forested regions where NHP density is greatest and who rely on bushmeat as a major food and income source. Our finding of 16 SFV WB-positive persons increases the total number of known global infections to 153, with 17 in DRC, including one woman who was previously found co-infected with HIV-1 and SFV in Kinshasa in 1985 [28]. These discoveries support the presence of SFV in humans in DRC for at least two decades in both urban and rural settings. Given the wide exposure to NHPs across DRC, our results suggest that SFV infection may have a wider geographic distribution within DRC. Our results also reinforce the potential for an increased SFV prevalence throughout the forested regions of Africa where humans and simians co-exist. Also, since SIV and STLV are highly prevalent in NHPs, rural populations in close contact with NHPs may also be at increased risk for co-infection with other simian retroviruses. Indeed, novel STLVs have been reported recently in NHP hunter cohorts from Cameroon who were also infected with SFV [8,29-31] suggesting that zoonotic transmission of simian retroviruses is an ongoing phenomenon. The continued clearing of forests combined with economic declines in DRC resulting from recurrent civil war forces residents to rely on alternative protein sources such as primate bushmeat and exacerbates the potential for increased exposure to simian retroviruses. Our study documents an increased risk of SFV infection in persons preparing NHPs for consumption, or getting bitten and scratched by NHPs, or bringing dead NHPs from the forest, though there was insufficient evidence to conclude elevated risk for SFV infection from hunting NHPs after controlling for age and gender in the multivariate models. However, we also found that low exposure activities, such as entrance into the forest daily, placed persons at elevated risk for SFV infection. SFV has been found in feces and urine and could be sources of infection in these frequent forest visits [14,32]. Nonetheless, direct evidence for such transmissions is not available. Human infection with SFV has been identified in animal handlers who did not report known mucocutaneous exposures to infected animals suggesting that less intimate contact with body fluids may possibly place persons at risk for infection [33]. While handling and processing of dead NHPs can lead to exposure to SFV and other simian retroviruses, contact with dead primates and other mammals in Africa has also resulted in fatal exposure and spread of the Ebola virus that causes hemorrhagic fever, raising public health concerns for this practice. Thus, increased public awareness is needed in these communities, including educational messages advising caution in primate hunting and handling, and forest safety, to prevent cross-species viral infections.

Curiously, SFV prevalence in our study population did not differ by gender. We have previously identified SFV antibodies in a female sex worker in DRC [28]. These results contrast with all other African studies where male hunters were more likely to be infected via more severe NHP contact, such as bite wounds [6,11,12]. Men who work with captive animals are also more frequently infected with SFV than women [6]. However, the higher prevalence seen in men in Cameroon and Gabon may have been biased by the recruitment of mostly hunters, which traditionally is a male activity in those countries. Data from our questionnaire support the fact that men are the primary hunters in DRC with women more commonly performing food preparation tasks. Although the number of severe cutaneous exposures in our study was low, this exposure increased the risk for SFV infection. Women processed bushmeat for consumption more frequently than men in our study possibly resulting in more mucocutaneous exposures. The reasons for our inability to detect SFV sequences in eleven WB-positive persons, including seven females and four males, are unclear and may be due to infection with highly divergent SFVs not detected with the current assays, low proviral loads, abortive infections, nonspecific seroreactivity, or other reasons. While similar results have been previously reported in other African countries which also used very generic PCR assays [11,12,24,25], the WB assay used in the current study has been shown to be highly sensitive and specific for detecting SFV infection [23]. Testing of longitudinal specimens may be necessary to investigate persistent SFV infection in these WB-positive and PCR-negative persons.

While the major route for SFV transmission in NHPs appears to be via saliva from bites by juvenile and adult animals [5,6], little is known about the transmission of SFV from mother-to-child. Nonetheless, vertical transmission should be considered, including during birth or through breast feeding since evidence for vertical transmission from an SFV-infected chimpanzee to a female offspring in captivity has been reported [33]. The youngest SFV-positive person in our study was an 11-year old girl who reported eating NHPs but not going into the forest, and her mother was not a study participant. Eating NHPs was not associated with WB-positivity in our study, thus, we are unable to assess the origin of her infection. Our study was primarily designed to enroll participants from each village in a serosurvey and thus included household contacts of persons found to be infected in each village, which facilitated our investigation of secondary SFV transmission. While we were unable to identify any serological evidence of SFV transmission to small numbers of close contacts tested in our study, the duration of the spousal relationships in this polygamous culture, the usage of barrier contraception that might prevent sexual transmission, and duration of breast feeding were not recorded to fully assess the effect of these factors on transmission. Proviral loads in one woman were high and were 12 times those reported in hunters from Cameroon, which ranged from undetectable to 145 copies/10^5^ cells [11]. The increased viral loads seen in this woman may result in increased transmissibility and/or pathogenicity of her infection but longitudinal studies are required to investigate this hypothesis. Although both of her teenage children were serologically negative for SFV, it is not known when the mother became infected. The wife of an SFV-infected hunter in Cameroon was reported to be persistently WB-positive but PCR-negative, suggestive of a possible case of person-to-person transmission [11]. However, information regarding her primate exposure was not disclosed to exclude a zoonotic origin of the infection. Likewise, we identified an SFV-infected woman in the current study who did not report any specific primate exposures also suggestive of a human-to-human transmission, but whose contacts were not available for testing to evaluate this possibility. Alternatively, self-reporting and recall bias could account for these results. Data on SFV shedding in vaginal secretions, breast milk, or semen from infected persons may provide important information on mother-to-child or sexual transmission.

The lower SFV prevalence seen in DRC, and the 1% reported in our study in Cameroon, contrasts with the 10.5% – 18.6% infection rate seen in Cameroon and Gabon in populations enriched for cross-species infections by targeting persons with severe simian exposures, including bites and wounds. The primary focus of our study was to investigate the seroprevalence of monkeypox infection in selected health zones in the Sankuru district and included persons with or without any NHP and animal exposure. Our study population reported few severe exposures and thus the lower SFV prevalence (0.5%) seen in our study most likely better represents infection prevalence in the general population compared to the higher rate seen in highly exposed persons in previous studies [11,12,24]. The lower SFV prevalence may also result from almost a third of our population being less than 10 years old, and younger children may have less overall NHP exposure. Interestingly, the SFV prevalence was about half that seen for HIV-1 (1.2%) in Central-south DRC reported from the 2007 Demographic and Health Survey (DHS) ( http://www.measuredhs.com). Unlike HIV-1, SFV is not screened for at blood banks and thus transmission via transfusion of contaminated blood products requires more thorough investigation. Blood-borne transmission has been reported in macaque models demonstrating that transmission via this route is possible [34,35].

Limited information prevents us from drawing strong conclusions on the disease potential of SFV infection [6]. Clinical information was not collected during our cross-sectional study so we were unable to ascertain possible diseases in SFV-infected persons. Longitudinal follow-up studies of infected persons and close contacts are required to better understand the epidemiology of these novel human infections, and to evaluate the role of SFV in disease and transmissibility. In addition, testing of persons with various diseases like cancer and neurological disorders in endemic regions would be beneficial to ascertain possible disease associations. Our data may also be limited by recall bias or the possible inaccurate reporting of NHP species that participants remembered exposure to. Although NHP species were chosen by participants using NHP pictures with their common names it may be difficult to distinguish between some monkey species.

Previously, we have shown that SFVs have co-evolved with their primate hosts over millions of years such that phylogenetic analysis of simian and human SFV sequences together can be used to accurately identify the species origin of human infections [3,6]. Detailed phylogenetic analysis using the largest dataset of SFV *pol* sequences assembled to date, including new SFVs endemic to NHPs from Cameroon and DRC, allowed exceptional resolution of the primate origin of human infections in DRC and also of those reported in Cameroon and Gabon [11,12]. We identified infection in three women in DRC with novel SFVs not seen before in humans, including SFV from red-tailed guenons (*Ce*. *ascanius*) and Angolan colobus (*Co*. *angolensis*), both of which inhabit only forests in DRC and are frequently hunted. Statistical analysis inferred that SFV prevalence was elevated in persons reporting exposure to Angolan colobus. In addition, we show that six hunters from Cameroon previously suspected of being infected with SFV from Debrazza guenons (*Ce*. *neglectus*) are actually infected with SFV from greater spot-nosed (*Ce*. *nictitans*) or moustached (*Ce*. *cephus*) monkeys, which are hunted more frequently than Debrazza monkeys in Cameroon [36]. Combined with SFV infection originating from red-tailed guenons, mona monkey (*Ce*. *mona*), and Debrazzas in persons from DRC, Cameroon and/or Gabon, humans are susceptible to SFV variants from five different *Cercopithecus* monkeys. Colobus, gorilla, chimpanzee, mandrill, baboon, and macaque SFVs can also infect humans from both captive and/or wild animals [6]. This high level of SFV diversity that has crossed into humans demonstrates a broad susceptibility to SFV infection, which contrasts with the more restricted susceptibility to SIV, since of the 45 known SIV variants only three (SIVcpz, SIVgor, SIVsmm) have crossed into humans at varying levels, two of which spread globally as HIV-1 (SIVcpz) and HIV-2 (SIVsmm). It is uncertain if any of the SFV variants identified so far will spread in humans and become a *bona fide* human virus.

## Conclusions

We report the results of a thorough epidemiologic investigation of SFV infection in DRC. Using a combined serologic and molecular approach we identify human infection with new SFVs from Angolan colobus and red-tailed monkeys, two NHP species hunted frequently in DRC. We also demonstrate that detailed phylogenetic analysis of SFVs found in humans, combined with those from simians in the same ecological niche, are needed to accurately define the zoonotic origin of human infections. Our findings suggest that SFV is widely prevalent across Africa in men and women exposed to NHPs and emphasize the importance of additional studies to determine human-to-human transmissibility and pathogenicity. The identification of endemic foci of SFV infection in women in three locations within central DRC will help facilitate these longitudinal studies.

## Methods

### Study population and specimen preparation

The population comprised 3,846 persons residing in 14 rural villages in the Sankuru district in the Kasai Oriental province in central DRC as part of a study on human monkeypox and other viral diseases (Figure 1). The local population includes subsistence farmers and hunters obtaining most of their protein from hunted wildlife, including monkeys, rodents, and ungulates. The most valued animal body parts are sold for economic sustenance, while the least nutritious parts are kept for personal consumption. The UCLA Institutional Review Board approved collection, storage, and future testing of blood samples collected in 2007 from all consenting study participants. Blood specimens were processed for plasma and buffy coats in DRC, aliquoted and stored at −80°C. A non-research determination was approved for testing of anonymized samples at CDC for retroviruses. Study questionnaires were completed to obtain demographic and animal contact information.

### NHP specimens and preparation

Dried blood spots (DBS) were collected from captive and hunted NHPs endemic to Cameroon and DRC, as previously described, with approvals from local zoos, and Forestry and Wildlife Ministries [37,38]. DNA was extracted from DBS using the Nuclisens (Biomeriuex) or Qiagen blood kits for the DRC and Cameroon samples, respectively, as described in detail elsewhere [37,39]. DNA integrity was confirmed by PCR of ß-actin sequences [37]. Species identification was recorded in the field and confirmed on all NHP samples from DRC by amplifying 12S rRNA sequences [40] or by phylogenetic analysis of SFV sequences as previously described [6].

### Simian foamy virus serology

Plasmas were first screened for SFV antibodies using a newly developed, high throughput, Luminex-based EIA assay. All seroreactive samples were then tested using a standard EIA format. Recombinant (rec) SFV proteins for the two EIA-based assays were prepared from complete SFV *gag* genes that were PCR-amplified from Cf2Th cultures infected with either African green monkey (SFVHu1) or chimpanzee (SFVHu6) SFVs previously isolated from humans [33]. Inclusion of proteins from both SFVHu1 and SFVHu6 in the serological assays ensures detection of broad seroreactivity to monkey and ape SFV, respectively [23]. Details of the Luminex-based EIA procedure have been reported recently [38]. Plasma was diluted 1:200 and incubated with the recGag proteins coupled to Luminex beads in microtiter plates. The BioPlex-200 (BioRad, Marnes La coquette, France) and BioPlex Software Manager v5.0 were used to analyze the reacted and washed bead sets. At least 100 events were read with results expressed as median fluorescence intensity (MFI)/100 beads. The cut-off value was calculated for each recGag protein as the mean MFI for all antibody negative reference sera plus 5 standard deviations and was set at 200 MFI. The sensitivity of the assay, evaluated on a reference panel of specimens from SFV WB-positive humans (n=47) and NHP species (n=13) was 85%. Assay specificity, assessed on a reference panel of 90 SFV WB-negative human samples was 97.2%.

Standard SFV EIA testing was performed with previously described methods using a 1:100 plasma dilution [25]. Each test run included SFVcpz and SFVagm-positive control plasmas and two pedigreed SFV-negative human control plasmas for assay validation. The EIA is 96% sensitive and 97% specific using human and simian plasma from PCR-positive (n=101) and PCR-negative (n-=605) individuals (data not shown). Seroreactivity was defined as those samples with an optical density (OD) greater than the cutoff of the mean OD values of the two negative controls plus 0.312, which is three standard deviations of the mean of the observed seroreactivity of the PCR-negative samples.

Specimens dually reactive in the Luminex and standard EIA were tested using a WB assay to confirm the observed seroreactivity as described previously [23]. Samples reactive to p68 and p72 or p70 and p74 Gag doublet proteins, while showing an absence of a similar reactivity to the uninfected CF2Th control antigen, were considered WB-positive and infected with SFV.

### SFV PCR, sequence analysis, and proviral loads

DNA was extracted from buffy coats available from persons with seropositive WB results using the Flexigene DNA extraction kit (Qiagen) and quantified using a Nanodrop instrument. DNA integrity was verified using β-actin PCR as described elsewhere [41,42]. One ug of DNA was input for a generic SFV nested PCR assay to detect 465-bp polymerase (*pol*) and 300-330-bp long terminal repeat (LTR) sequences as described elsewhere [23,25]. The *pol* and LTR primers can detect highly divergent SFV from a variety of primate species [6,23]. DNA from tissue culture cells infected with a macaque SFV (SFVmac) was used as a positive control for both PCRs. SFV *pol* sequences were aligned using Clustal W along with reference and new sequences representative of a number of NHP species, including those endemic to DRC, Cameroon, and Gabon. Phylogenies were constructed using neighbor-joining (NJ), maximum likelihood (ML), and Bayesian inference as described previously [3,6,26]. Briefly, NJ and ML methods were implemented using the program MEGA v5.0 [43]. The HKY+G (0.8518) model of nucleotide substitution was inferred using the ML method with goodness of fit measured by the Bayesian information criterion in MEGA5 for the NJ and ML methods. 1,000 bootstrap replicates were used to determine the robustness of the NJ and ML tree topologies. Bayesian trees were inferred using the program BEAST v1.6.2 and the HKY+G model with an uncorrelated lognormal relaxed-clock model of nucleotide substitution and a Yule speciation tree prior and 40 million Markov chain Monte Carlo (MCMC) chains [44]. Convergence of the chain sampling was checked in the program Tracer for effective sample sizes (ESS) > 500. Trees were saved every 4,000 generations and the tree with the maximum product of the posterior clade probabilities (maximum clade credibility tree) was chosen from the posterior distribution of 10,001 sampled trees after burning in the first 1,000 sampled trees with the program TreeAnnotator version 1.6.2. Trees were viewed in FigTree version 1.3.1 and in the MEGA5 tree editor.

Limited information exists regarding viral loads in SFV-infected humans and NHPs [11], which has been associated with increased pathogenicity and transmissibility of other human retroviruses. To investigate proviral loads in PCR-positive persons we developed a generic quantitative PCR test (qPCR) that detects *pol* sequences using primers SIF4O (5’CTMACTAGTATTGCWRTTCCRAAGGT3’) and SIR1N (5’GTTTTATITCACTATTTTTCCTTTCCAC3’), and probes SIP4O (FAM5’CACTCYGATYARFFKRCDGCATTCAC3’BHQ1) and SIP5RON (FAM5’CTTTGRGGRTGRTAAGGAGTACTGWATTCC3’BHQ1). One μg DNA, equivalent to about 10^5^ lymphocytes, from PCR-positive persons was tested using the AmpliTaq Gold system (Applied Biosystems/Roche, Branchtown, New Jersey) on a BioRad CFX96 iCycler (Hercules, CA) for 95°C for 10 min, followed by 55 cycles of 95°C and 52°C for 15 sec each, and 62°C for 30 sec. Standard control templates were engineered from *pol* sequences from SFV-infected humans, NHPs, or cell lines with primers SIF3 and SIR3 [26] or DNHP2N (5’CCTTTTGATAAATTYTWATTGAYTATATTGG3’) and FVPGR2 (5’CCCTTTGCAAACCCCGATACACTTGACTTC3’) and cloned into pCR2.1 TOPO (Invitrogen, Carlsbad, CA). To ensure broad reactivity of the assay for detecting monkey and ape SFVs, control templates were generated from known SFV-positive persons from Cameroon (2719YI, 1465MV, 1083MO, and 2467LE) infected with SFV variants from mona monkey (*Ce*. *mona*), mandrill (*Mandrillus sphinx*), gorilla (*Gorilla gorilla*), and greater spot-nosed monkey (*Ce*. *nictitans*) [25], respectively, a worker (199917) and chimpanzee (X174) infected with chimpanzee SFV (SFVcpz) [33], and cell lines infected with macaque (SFVmac), orangutan (SFV11), baboon (SFV10), or African green monkey (SFV3) SFVs.

### Statistical methods

All collected information for the study population of 3,846 persons was used in estimation of SFV prevalence, frequency and percentage with exposure to NHPs, and assessment of epidemiological associations with SFV infection. Non-monotonically missing data included 512 (13.3%) persons with missing plasma for serological testing data, 850 (22.1%) missing exposure survey data, and 106 (2.8%) missing demographic data. The data were assumed to be missing at random for implementation of multiple imputation methods [45]. All variables used in the analyses, in addition to auxiliary variables that may be associated with variables having missing data, were included in the multivariate normal imputation model as binary-coded variables. A MCMC method of multiple imputation replaced each missing value with a set of plausible values that represent the uncertainty about the unknown values. Diagnostic tests were performed to examine stability and convergence. Ten complete data sets were then analyzed using standard procedures and the results were combined from these analyses. Results from multiply imputed complete datasets were compared to those estimated from incomplete data to demonstrate close reproduction of relationships in the data (data not shown).

Variables indicating exposure to various NHP species or activity (e.g. hunting, cooking, etc.) that led to exposure were created by combining binary responses to primate exposure-specific survey questions (e.g. hunting *Co*. *angolensis*, cooking *Co*. *angolensis*, etc.). Created NHP exposure variables are binary; exposure activity variables are discrete, summing the number of different species that were encountered for a specific activity. Because these two types of exposure variables (NHP species and activity) were created from the same survey questions, multicollinearity exists between these variables. For this reason, two distinct multivariable regression models were implemented to assess factors associated with SFV infection, one that included variables for exposure to NHP species, the other including NHP exposure activities. The NHP exposure activity variables were further refined to reflect results from the model of SFV risk associated with NHP species. Of the ten NHP species or species groups, the five with the greatest SFV prevalence were equally weighted to that of the other five lower prevalence species groups combined; the discrete exposure activity variables therefore have possible outcomes ranging from 0 to 6. Multivariable and single variable logistic regression models of SFV prevalence were implemented to assess association with the demographic variables age and gender, daily visits to the forest, and NHP exposure related activities or exposure to species. Odds ratios and 95% confidence limits were calculated.

## Competing interests

The authors declare that they have no competing interests.

## Authors’ contributions

WMS, MP, ML, and AWR conceived and designed the study. NDW, ML, CFD, UT, JJM-T, EOW, PM, MP, and AWR provided specimens and data on study population. AA and AE, and HZ developed and applied the SFV Luminex-based EIA and qPCR test, respectively. ST, AS, HZ, SA-M, and AA performed specimen testing and data analysis with WMS and MP. DLH performed the statistical analyses. WMS, AS, DLH, WH, MP, and AWR wrote the manuscript. WMS, ML, NH, and PM analyzed the questionnaire data. All authors read and approved the final manuscript.

## References

[B1] Sharp PM Hahn BH The evolution of HIV-1 and the origin of AIDS Philos Trans R Soc Lond B Biol Sci 2010 365 2487 2494 10.1098/rstb.2010.0031 20643738 PMC2935100

[B2] Wolfe ND Switzer WM Heneine W Scheld DCH WM, Hughes JM Emergence of novel retroviruses Emerging Infections 2006 1st Washington DC: ASM Press 139 152

[B3] Switzer WM Salemi M Shanmugam V Gao F Cong ME Kuiken C Bhullar V Beer BE Vallet D Gautier-Hion A Ancient co-speciation of simian foamy viruses and primates Nature 2005 434 376 380 10.1038/nature03341 15772660

[B4] Meiering CD Linial ML Historical perspective of foamy virus epidemiology and infection Clin Microbiol Rev 2001 14 165 176 10.1128/CMR.14.1.165-176.2001 11148008 PMC88968

[B5] Murray SM Linial ML Foamy virus infection in primates J Med Primatol 2006 35 225 235 10.1111/j.1600-0684.2006.00171.x 16872286

[B6] Switzer WM Heneine W Liu D Foamy virus infection of humans Molecular Detection of Human Viral Pathogens Volume 1 2011 Boca Raton: CRC Press, Taylor & Francis Group 131 146

[B7] Khan AS Simian foamy virus infection in humans: prevalence and management Expert Rev Anti Infect Ther 2009 7 569 580 10.1586/eri.09.39 19485797

[B8] Wolfe ND Heneine W Carr JK Garcia AD Shanmugam V Tamoufe U Torimiro JN Prosser AT Lebreton M Mpoudi-Ngole E Emergence of unique primate T-lymphotropic viruses among central African bushmeat hunters Proc Natl Acad Sci U S A 2005 102 7994 7999 10.1073/pnas.0501734102 15911757 PMC1142377

[B9] Lerche NW Switzer WM Yee JL Shanmugam V Rosenthal AN Chapman LE Folks TM Heneine W Evidence of infection with simian type D retrovirus in persons occupationally exposed to nonhuman primates J Virol 2001 75 1783 1789 10.1128/JVI.75.4.1783-1789.2001 11160676 PMC114087

[B10] Khabbaz RF Heneine W George JR Parekh B Rowe T Woods T Switzer WM McClure HM Murphey-Corb M Folks TM Brief report: infection of a laboratory worker with simian immunodeficiency virus N Engl J Med 1994 330 172 177 10.1056/NEJM199401203300304 8264739

[B11] Betsem E Rua R Tortevoye P Froment A Gessain A Frequent and recent human acquisition of simian foamy viruses through apes' bites in central Africa PLoS Pathog 2011 7 e1002306 10.1371/journal.ppat.1002306 22046126 PMC3203161

[B12] Mouinga-Ondeme A Caron M Nkoghe D Telfer P Marx P Saib A Leroy E Gonzalez JP Gessain A Kazanji M Cross-species transmission of simian foamy virus to humans in rural Gabon, central Africa J Virol 2011 86 1255 1260 22072747 10.1128/JVI.06016-11 PMC3255803

[B13] Huang F Wang H Jing S Zeng W Simian foamy virus prevalence in Macaca mulatta and zookeepers AIDS Res Hum Retroviruses 2012 28 591 593 10.1089/aid.2011.0305 22236106

[B14] Boneva RS Switzer WM Spira TJ Bhullar VB Shanmugam V Cong ME Lam L Heneine W Folks TM Chapman LE Clinical and virological characterization of persistent human infection with simian foamy viruses AIDS Res Hum Retroviruses 2007 23 1330 1337 10.1089/aid.2007.0104 18184074

[B15] Wolfe ND Daszak P Kilpatrick AM Burke DS Bushmeat hunting, deforestation, and prediction of zoonoses emergence Emerg Infect Dis 2005 11 1822 1827 10.3201/eid1112.040789 PMC3367616 16485465

[B16] Woolhouse ME Population biology of emerging and re-emerging pathogens Trends Microbiol 2002 10 S3 S7 10.1016/S0966-842X(02)02428-9 12377561

[B17] Ahuka-Mundeke S Mbala-Kingebeni P Liegeois F Ayouba A Lunguya-Metila O Demba D Bilulu G Mbenzo-Abokome V Inogwabini BI Muyembe-Tamfum JJ Identification and Molecular Characterization of New Simian T Cell Lymphotropic Viruses in Nonhuman Primates Bushmeat from the Democratic Republic of Congo AIDS Res Hum Retroviruses 2011 28 628 635 21827287 10.1089/aid.2011.0211 PMC3358107

[B18] Peeters M Courgnaud V Abela B Auzel P Pourrut X Bibollet-Ruche F Loul S Liegeois F Butel C Koulagna D Risk to human health from a plethora of simian immunodeficiency viruses in primate bushmeat Emerg Infect Dis 2002 8 451 457 10.3201/eid0805.010522 11996677 PMC2732488

[B19] Smith KM Anthony SJ Switzer WM Epstein JH Seimon T Jia H Sanchez MD Huynh TT Galland GG Shapiro SE Zoonotic viruses associated with illegally imported wildlife products PLoS One 2012 7 e29505 10.1371/journal.pone.0029505 22253731 PMC3254615

[B20] Niama FR Toure-Kane C Vidal N Obengui P Bikandou B Ndoundou Nkodia MY Montavon C Diop-Ndiaye H Mombouli JV Mokondzimobe E HIV-1 subtypes and recombinants in the Republic of Congo Infect Genet Evol 2006 6 337 343 10.1016/j.meegid.2005.12.001 16473564

[B21] Vidal N Mulanga C Bazepeo SE Mwamba JK Tshimpaka JW Kashi M Mama N Laurent C Lepira F Delaporte E Peeters M Distribution of HIV-1 variants in the Democratic Republic of Congo suggests increase of subtype C in Kinshasa between 1997 and 2002 J Acquir Immune Defic Syndr 2005 40 456 462 10.1097/01.qai.0000159670.18326.94 16280702

[B22] Vidal N Peeters M Mulanga-Kabeya C Nzilambi N Robertson D Ilunga W Sema H Tshimanga K Bongo B Delaporte E Unprecedented degree of human immunodeficiency virus type 1 (HIV-1) group M genetic diversity in the Democratic Republic of Congo suggests that the HIV-1 pandemic originated in Central Africa J Virol 2000 74 10498 10507 10.1128/JVI.74.22.10498-10507.2000 11044094 PMC110924

[B23] Hussain AI Shanmugam V Bhullar VB Beer BE Vallet D Gautier-Hion A Wolfe ND Karesh WB Kilbourn AM Tooze Z Screening for simian foamy virus infection by using a combined antigen Western blot assay: evidence for a wide distribution among Old World primates and identification of four new divergent viruses Virology 2003 309 248 257 10.1016/S0042-6822(03)00070-9 12758172

[B24] Calattini S Betsem EB Froment A Mauclere P Tortevoye P Schmitt C Njouom R Saib A Gessain A Simian foamy virus transmission from apes to humans, rural Cameroon Emerg Infect Dis 2007 13 1314 1320 10.3201/eid1309.061162 18252101 PMC2857270

[B25] Wolfe ND Switzer WM Carr JK Bhullar VB Shanmugam V Tamoufe U Prosser AT Torimiro JN Wright A Mpoudi-Ngole E Naturally acquired simian retrovirus infections in central African hunters Lancet 2004 363 932 937 10.1016/S0140-6736(04)15787-5 15043960

[B26] Goldberg TL Sintasath DM Chapman CA Cameron KM Karesh WB Tang S Wolfe ND Rwego IB Ting N Switzer WM Coinfection of Ugandan red colobus (Procolobus [Piliocolobus] rufomitratus tephrosceles) with novel, divergent delta-, lenti-, and spumaretroviruses J Virol 2009 83 11318 11329 10.1128/JVI.02616-08 19692478 PMC2772775

[B27] Engel G Hungerford LL Jones-Engel L Travis D Eberle R Fuentes A Grant R Kyes R Schillaci M Risk assessment: a model for predicting cross-species transmission of simian foamy virus from macaques (M. fascicularis) to humans at a monkey temple in Bali, Indonesia Am J Primatol 2006 68 934 948 10.1002/ajp.20299 16900504

[B28] Switzer WM Garcia AD Yang C Wright A Kalish ML Folks TM Heneine W Coinfection with HIV-1 and simian foamy virus in West Central Africans J Infect Dis 2008 197 1389 1393 10.1086/587493 18444796

[B29] Calattini S Betsem E Bassot S Chevalier SA Mahieux R Froment A Gessain A New strain of human T lymphotropic virus (HTLV) type 3 in a Pygmy from Cameroon with peculiar HTLV serologic results J Infect Dis 2009 199 561 564 10.1086/596206 19099485

[B30] Calattini S Chevalier SA Duprez R Bassot S Froment A Mahieux R Gessain A Discovery of a new human T-cell lymphotropic virus (HTLV-3) in Central Africa Retrovirology 2005 2 30 10.1186/1742-4690-2-30 15882466 PMC1142341

[B31] Zheng H Wolfe ND Sintasath DM Tamoufe U Lebreton M Djoko CF Diffo Jle D Pike BL Heneine W Switzer WM Emergence of a novel and highly divergent HTLV-3 in a primate hunter in Cameroon Virology 2010 401 137 145 10.1016/j.virol.2010.03.010 20353873 PMC2862145

[B32] Liu W Worobey M Li Y Keele BF Bibollet-Ruche F Guo Y Goepfert PA Santiago ML Ndjango JB Neel C Molecular ecology and natural history of simian foamy virus infection in wild-living chimpanzees PLoS Pathog 2008 4 e1000097 10.1371/journal.ppat.1000097 18604273 PMC2435277

[B33] Switzer WM Bhullar V Shanmugam V Cong ME Parekh B Lerche NW Yee JL Ely JJ Boneva R Chapman LE Frequent simian foamy virus infection in persons occupationally exposed to nonhuman primates J Virol 2004 78 2780 2789 10.1128/JVI.78.6.2780-2789.2004 14990698 PMC353775

[B34] Brooks JI Merks HW Fournier J Boneva RS Sandstrom PA Characterization of blood-borne transmission of simian foamy virus Transfusion 2007 47 162 170 10.1111/j.1537-2995.2007.01079.x 17207245

[B35] Khan AS Kumar D Simian foamy virus infection by whole-blood transfer in rhesus macaques: potential for transfusion transmission in humans Transfusion 2006 46 1352 1359 10.1111/j.1537-2995.2006.00862.x 16934071

[B36] Aghokeng AF Ayouba A Mpoudi-Ngole E Loul S Liegeois F Delaporte E Peeters M Extensive survey on the prevalence and genetic diversity of SIVs in primate bushmeat provides insights into risks for potential new cross-species transmissions Infect Genet Evol 2010 10 386 396 10.1016/j.meegid.2009.04.014 19393772 PMC2844463

[B37] Sintasath DM Wolfe ND Lebreton M Jia H Garcia AD Le Doux-Diffo J Tamoufe U Carr JK Folks TM Mpoudi-Ngole E Simian T-lymphotropic virus diversity among nonhuman primates Cameroon. Emerg Infect Dis 2009 15 175 184 10.3201/eid1502.080584 PMC2657614 19193260

[B38] Ahuka-Mundeke S Ayouba A Mbala-Kingebeni P Liegeois F Esteban A Lunguya-Metila O Demba D Bilulu G Mbenzo-Abokome V Inogwabini BI Novel multiplexed HIV/simian immunodeficiency virus antibody detection assay Emerg Infect Dis 2011 17 2277 2286 10.3201/eid1712.110783 22172157 PMC3311211

[B39] Liegeois F Lafay B Switzer WM Locatelli S Mpoudi-Ngole E Loul S Heneine W Delaporte E Peeters M Identification and molecular characterization of new STLV-1 and STLV-3 strains in wild-caught nonhuman primates in Cameroon Virology 2008 371 405 417 10.1016/j.virol.2007.09.037 17976676

[B40] van der Kuyl AC Kuiken CL Dekker JT Goudsmit J Phylogeny of African monkeys based upon mitochondrial 12S rRNA sequences J Mol Evol 1995 40 173 180 10.1007/BF00167111 7535363

[B41] Switzer WM Pieniazek D Swanson P Samdal HH Soriano V Khabbaz RF Kaplan JE Lal RB Heneine W Phylogenetic relationship and geographic distribution of multiple human T-cell lymphotropic virus type II subtypes J Virol 1995 69 621 632 7815525 10.1128/jvi.69.2.621-632.1995 PMC188622

[B42] Switzer WM Michler RE Shanmugam V Matthews A Hussain AI Wright A Sandstrom P Chapman LE Weber C Safley S Lack of cross-species transmission of porcine endogenous retrovirus infection to nonhuman primate recipients of porcine cells, tissues, or organs Transplantation 2001 71 959 965 10.1097/00007890-200104150-00022 11349732

[B43] Tamura K Peterson D Peterson N Stecher G Nei M Kumar S MEGA5: Molecular Evolutionary Genetics Analysis using Maximum Likelihood, Evolutionary Distance, and Maximum Parsimony Methods Mol Biol Evol 2011 28 2731 2739 10.1093/molbev/msr121 21546353 PMC3203626

[B44] Drummond AJ Rambaut A BEAST: Bayesian evolutionary analysis by sampling trees BMC Evol Biol 2007 7 214 10.1186/1471-2148-7-214 17996036 PMC2247476

[B45] Schafer JL Analysis of Incomplete Multivariate Data 1997 London: Chapman & Hall

